# Human Mesh Reconstruction with Generative Adversarial Networks from Single RGB Images

**DOI:** 10.3390/s21041350

**Published:** 2021-02-14

**Authors:** Rui Gao, Mingyun Wen, Jisun Park, Kyungeun Cho

**Affiliations:** Department of Multimedia Engineering, Dongguk University-Seoul, 30, Pildongro-1-gil, Jung-gu, Seoul 04620, Korea; gaorui@dongguk.edu (R.G.); wmy_dongguk@dongguk.edu (M.W.); jisun@dongguk.edu (J.P.)

**Keywords:** artificial intelligence, image processing, GAN, deep learning, 3D human model, smart cities

## Abstract

Applications related to smart cities require virtual cities in the experimental development stage. To build a virtual city that are close to a real city, a large number of various types of human models need to be created. To reduce the cost of acquiring models, this paper proposes a method to reconstruct 3D human meshes from single images captured using a normal camera. It presents a method for reconstructing the complete mesh of the human body from a single RGB image and a generative adversarial network consisting of a newly designed shape–pose-based generator (based on deep convolutional neural networks) and an enhanced multi-source discriminator. Using a machine learning approach, the reliance on multiple sensors is reduced and 3D human meshes can be recovered using a single camera, thereby reducing the cost of building smart cities. The proposed method achieves an accuracy of 92.1% in body shape recovery; it can also process 34 images per second. The method proposed in this paper approach significantly improves the performance compared with previous state-of-the-art approaches. Given a single view image of various humans, our results can be used to generate various 3D human models, which can facilitate 3D human modeling work to simulate virtual cities. Since our method can also restore the poses of the humans in the image, it is possible to create various human poses by given corresponding images with specific human poses.

## 1. Introduction

Simulations and test platforms for smart cities require various human meshes to achieve a realistic depiction of the virtual world. For the convenience of testing algorithms of smart city, a virtual city as a test platform is necessary. The virtual city should be realistic to the real city, for example, various kind of models for humans, buildings, vehicles, etc. should be simulated. The pose of human should also be considered when analyzing the human behavior in smart cities. For example, a human with a hand reaching out towards the street indicates the human wants to call a taxi, in this case, the simulated AI taxi should analysis the human pose and stop to wait for the human get into the car. Conventional modeling methods such as using 3D modeling software consume a lot of time. To facilitate the modeling work, more efficient methods should be invented. Generation of human meshes and poses by processing the data captured by various sensors has been studied [1,2,3]. Currently, the primary method to obtain a high-quality human mesh is based on light detection and ranging (LiDAR) [1] or depth cameras [2], by 3D scanning the entire human body; however, these devices are expensive, bulky, and difficult to transport. Consequently, some studies have proposed the reconstruction of the human body mesh by using a multi-camera system [3]; however, the construction of such systems is cumbersome. Not only do the camera positions need to be calibrated but the cameras also need to be synchronized. In addition, the cost of reconstructing the system is high due to the considerable investment for the cameras themselves, which limits its application and the promotion of multi-camera human mesh reconstruction systems. Owing to the increasing improvements in the imaging quality of mobile devices (such as mobile phones), significant research efforts have been devoted toward obtaining high-quality 3D human meshes using a single RGB image captured by a camera [4]. The objective of this study is to obtain an accurate human body mesh from a single image using deep learning methods, in order to facilitate the creation of a variety of human body meshes. The meshes generated via the proposed method can be easily updated using other tools such as texture. The presented method efficiently generates human meshes from single images; these meshes can be used in smart city simulations after postprocessing to obtain a rich variety of human meshes for various simulations and reduce the cost of creating smart cities. Additionally, the proposed method in this paper can acquire the 3D pose data of the human body while acquiring the mesh of the human model. It can be used in human behavior recognition (HBR) [5] and planning human-oriented, pedestrian-friendly intersections in smart cities [6].

A previous study [7] proposed a novel method to reconstruct 3D human meshes. This method generates a 3D human mesh through the conditional generation confrontation mode, with unpaired 2D key notes and 3D scans. Given an image, the network infers 3D mesh parameters and perspectives of the camera, such that the 3D keypoints match the annotated 2D keypoints after projection. These parameters are then sent to a discriminator network, which is tasked with determining whether the 3D parameters correspond to the features of real people. This approach can generate richer and more useful mesh representations, as compared to most current methods [8,9], for calculating 2D or 3D joint positions. The main step in this approach is the minimization of the reprojection loss of keypoints, which allows for the mesh to be trained with images containing only the ground truth (GT) 2D annotations.

However, the performance in 3D human reconstruction in the wild is still not satisfactory, as only the features of single pictures are extracted and regressed to the 3D mesh parameters. Moreover, only a dataset with 2D bone annotation is used, which also affects the accuracy of details in the 3D human reconstruction.

Accordingly, this study proposes a generative adversarial network (GAN) to generate a 3D human mesh considering both shape and pose accuracy, by using a shape–pose-based generator and a multi-source discriminator trained over multiple types of datasets.

As deep convolutional neural networks (DCNNs) are powerful, significant progress has been achieved in the estimation of 3D human posture using monocular images. To improve the accuracy of shape reconstruction, this study adopts a newly designed shape–pose-based generator (based on the DCNN) and an enhanced multi-source discriminator. The generator consists of three parts: an encoding 2D pose module, an encoding shape module, and a fitting 3D parametric module. The multi-source discriminator promotes adversarial learning, and it considers four key factors: (1) the description of image–pose correspondence; (2) the description of image–shape correspondence; (3) the constraints on human joints; and (4) articulation constraint of the human body. This technology can achieve state-of-the-art performance in terms of both quality and quantity.

The main contributions of this study are summarized as follows:

- An adversarial learning method is proposed for extracting 3D meshes from 2D images.
- A novel multi-source discriminator is designed to enhance the generalization ability of the generator.
- The accuracy of the shape is improved when reconstructing human models.

The remainder of this paper is organized as follows: Section 2 summarizes several previous studies and the work done therein. Section 3 describes the proposed method in detail, and Section 4 presents the results and analysis of the conducted experiments. Finally, Section 5 presents the concluding remarks.

## 2. Related Work

3D reconstruction has been widely studied and applied in various fields. This section introduces existing state-of-the-art approaches for human 3D reconstruction and compares these approaches.

A volumetric regression network (VRN) has been used for 3D face reconstruction [10]. Aaron S. et al. improved the VRN for human 3D reconstruction and proved that the enhanced VRN could reconstruct the 3D human mesh by means of training the network with a suitable dataset. In addition, it could generate a 3D human mesh with more complex poses using a given set of high-quality training data with just a single image as the input. However, it was trained using a generated dataset. Hence, the performance declined when it was applied to a real dataset.

Dense correspondences were proposed to describe the relationship between an RGB image and the human body surface in the approach by Rıza et al. [11]. The location of each pixel was determined on a map, and 2D correction was applied. In another study, feature pyramid and region-based convolutional neural networks were used to generate human 3D meshes from a 2D RGB image. A teacher net was used to assist in training, which was by means of a fully convolutional neural network.

A biomechanically inspired recurrent neural network (Bio-LSTM) is a recursive neural network developed based on biomechanics [6]. It can predict the pose of 3D joints and the position of a person in a global coordinate system. The network could also simultaneously predict the posture and global positions of multiple persons and was effective up to a distance of 45 m (between the human and the camera). The output of the network was a 3D mesh of the entire body expressed using the parameters of the skinned multi-person linear (SMPL) model. Moreover, a new objective function was proposed to ensure aspects such as the periodicity of human walking and the mirror symmetry of the human body. However, this approach focused only on the standing and walking poses and could not distinguish between males and females.

Compared to previous approaches for 3D human reconstruction, human mesh recovery (HMR) based on the position of 2D or 3D joints could generate a richer and more useful mesh of the human body [7]. This approach minimized reprojection losses at key points, which enabled the network to be trained using images with only the GT 2D annotations. In addition, a trained discriminator was added, such that it could assess whether the generated 3D human model was similar to reality on the basis of the 3D human mesh database. However, the 2D annotation was not sufficient to accurately generate the 3D human model.

In a different study, semantic segmentation was proven to be an effective approach for human 3D reconstruction [12]. Hossain et al. proposed an approach to generate a 3D human model with two steps. First, the 2D pose was estimated from 2D RGB images using an advanced 2D pose estimator, after which the 2D pose was mapped onto the 3D space. Subsequently, a time-series of the 2D positions of joints was used to estimate the 3D human pose time-based sequence to avoid the jitter caused by independent errors in each frame. However, only one type of feature was used to train the network. Nonetheless, it can be extended to other features to improve the approach significantly.

Overall, to estimate the human pose and shape from images, recent studies have proposed learning based on thousands of scans of real human body models, typically parameterized using individual body poses and shapes [1,13,14]. Specifically, convolutional neural networks (CNNs) can predict the parameters of the SMPL 3D body model from images [13] and then reproject the model to the image to evaluate the loss function in the 2D space. Thus, 2D pose annotations can be used to train such architectures.

GAN, originally proposed in [15], has been employed to generate images of the human body in arbitrary poses [16]; a new approach based on the SMPL parameters for generating human models was proposed [7]. Moreover, models for modeling continuous face animations were presented; the GAN method was also used to edit and generate a face that could talk [17,18,19].

Table 1 lists the features of different frameworks. By comparison, our framework advances the frameworks [6,9] by being capable recovering meshes of humans and running in real time. Our framework is more similar to that in [7]. The outputs are compared in section of Experimental Analysis.

## 3. Human Mesh Reconstruction Using a Single Image 

### 3.1. Overview of Proposed Method

**Figure 1:**
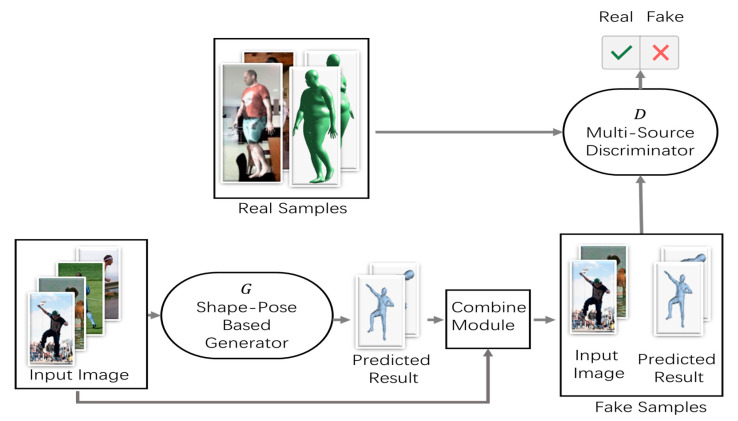
Overview of Proposed Method.

This paper proposes an approach to reconstruct a human mesh using a single image; the human mesh is used to measure the body shape automatically by means of deep learning methods. Figure 1 illustrates the human body mesh reconstruction process based on the proposed deep learning approach for human body shape estimations. To improve the accuracy of posture estimation along with the accuracy of human body shape estimation, this study adopts the newly designed shape–pose-based generator (based on a DCNN) and an enhanced multi-source discriminator. Figure 1 presents the entire process of the proposed approach.

The proposed method utilizes the GAN structure. In the first step, based on the input image information, a 3D human model is generated using the shape–pose-based generator. The generator was designed based on a stacked hourglass network [20] that can effectively extract image information to predict the key points and shapes of the human body. Next, the 3D human model and images are fed into the multi-source discriminator simultaneously. The pose–shape-based generator generates the results by learning the GT 3D annotations, such that the discriminator cannot distinguish between the real 3D mesh and the predicted mesh.

The 3D mesh generator, *G*, is trained to generate samples $S_{sample}\left(I_{n},M\left(P_{n}^{3D},S_{n}\right)\right),$ where $I_{n}$ is the input image and $M\left(P_{n}^{3D},S_{n}\right)$ is the mesh parameter, including $P_{n}^{3D}$, the pose information, and $S_{n}$, the shape information, in a manner that confuses the discriminator, *D*, which, in turn, attempts to distinguish them from real samples ${\hat{s}}_{sample}\left(I_{n},\hat{M}\left({\hat{P}}_{n}^{3D},{\hat{S}}_{n}\right)\right),$ where $\hat{M}\left({\hat{P}}_{n}^{3D},{\hat{S}}_{n}\right)$ is the real mesh parameter. In the method proposed in this paper, the generator attempts to trick the discriminator by predicting the exact 3D posture and shape. The discriminator distinguishes the real 3D pose and shape from the predicted pose and shape. As the predicted mesh can be generated from images captured in a laboratory environment (with 3D annotations) as well as unannotated images in the wild, the human structure learned from the 3D dataset can be adapted to in-the-wild images through adversarial learning.

### 3.2. Shape–Pose-Based Generator

**Figure 2:**
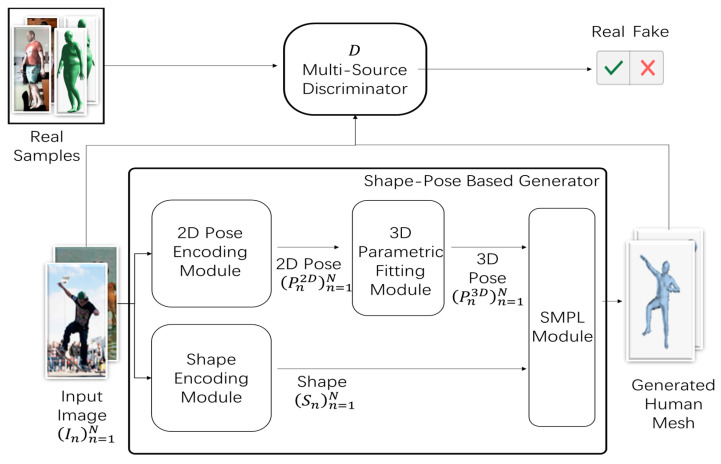
Overview of Proposed Generator.

Figure 2 illustrates the shape–pose-based generator process in detail. Let ${\left(I_{n}\right)}_{n=1}^{N}$ denote the input image, where N denotes the image indexes. ${\left(P_{n}^{2D}\right)}_{n=1}^{N}$ denotes the result of encoding the 2D pose module, where $P^{2D}\in R^{3K}$ is modeled using the keypoints’ position with K=15 keypoints. ${\left(S_{n}^{3D}\right)}_{n=1}^{N}$ denotes the result of encoding the shape module, where $S\in R^{10}$ is obtained via parameterization of the first ten coefficients of the principle component analysis (PCA) shape space. ${\left(P_{n}^{3D}\right)}_{n=1}^{N}$ denotes the result of fitting the 3D parametric module, where $P^{3D}\in R^{3K}$ is modeled using the keypoints’ position with K=15 keypoints.

The generator consists of the 2D pose encoding module, the shape encoding module, the 3D parametric fitting module, and the SMPL module, as shown in Figure 2. First, ${\left(I_{n}\right)}_{n=1}^{N}$ is input into the encoding 2D pose module and the encoding shape module and the parameters of 2D pose ${\left(P_{n}^{2D}\right)}_{n=1}^{N}$ and shape ${\left(S_{n}\right)}_{n=1}^{N}$ are obtained, respectively. Subsequently, the obtained parameters ${\left(P_{n}^{2D}\right)}_{n=1}^{N}$ are fed to the fitting 3D parametric module to obtain the 3D parameters ${\left(P_{n}^{3D}\right)}_{n=1}^{N}$. Finally, the obtained 3D parameters ${\left(P_{n}^{3D}\right)}_{n=1}^{N}$ and ${\left(S_{n}\right)}_{n=1}^{N}$ are fed into the SMPL module to generate the 3D human mesh.

The SMPL module is a network for generating bone-driven mesh. Bone-driven mesh can accurately represent the various shapes of the body’s natural state, which deforms with posture. If parameters from a large number of datasets are learned, the reconstruction errors can be minimized to create a mesh as close to reality as possible. With this network, the mesh can be quickly rendered and easily deployed. Moreover, the mesh would also be compatible with existing rendering engines.

### 3.3. Multi-Source Discriminator

**Figure 3:**
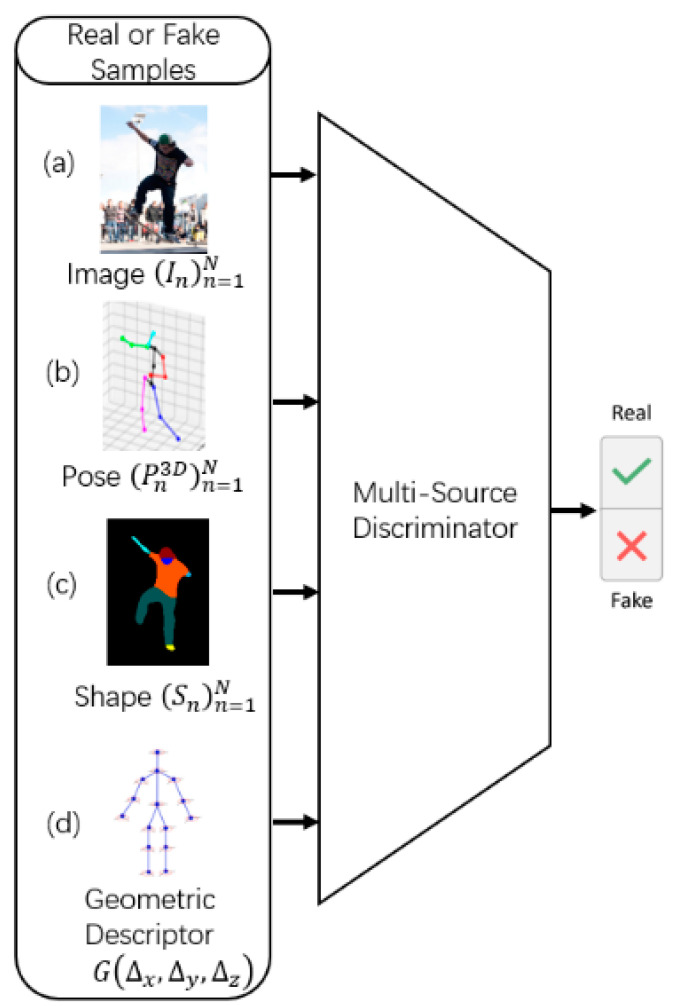
Overview of Proposed Discriminator.

The poses predicted by the generator from both the 3D pose dataset and the in-the-wild images are treated as “fake” examples for training the discriminator. During the adversarial learning phase, the pose–shape-based generator generates results by learning the GT 3D annotations such that the discriminator cannot distinguish between the real 3D mesh and the predicted mesh. Consequently, for in-the-wild images without annotation, the method proposed herein also performs the corresponding prediction; hence, the prediction results are similar to the distribution of images with real 3D annotations. Although unannotated in-the-wild images are difficult to use directly for training the generator, their corresponding results can be used as “fake” examples for training discriminators in order to help tune the generator. The discriminator determines whether the estimated result is similar to the GT. the quality of the discriminator impacts the quality of the generator; hence, a multi-source network architecture is designed here. Figure 3 illustrates the multi-source discriminator process in detail.

In the discriminator (shown in Figure 3), there are four information sources: (1) the original image ${\left(I_{n}\right)}_{n=1}^{N}$; (2) the position of the keypoints ${\left(P_{n}^{3D}\right)}_{n=1}^{N}$; (3) the body shape information ${\left(S_{n}\right)}_{n=1}^{N}$; and (4) the pairwise relative locations and distances ($G\left(\Delta x,\Delta y,\Delta z\right)(\Delta x=x_{i}-x_{j},\Delta y=y_{i}-y_{j},\Delta z=z_{i}-z_{j}$, where $\left(x_{i},y_{i},z_{i}\right)$ and $\left(x_{j},y_{j},z_{j}\right)$ denote the 3D position of the body keypoints i and j, respectively).

The information source considers three key factors: (1) description of image–posture correspondence; (2) description of the corresponding image–shape relationship; and (3) human body articulation constraints.

To model the image-mesh correspondence, this study uses the original image ${\left(I_{n}\right)}_{n=1}^{N}$ as the first source of information, which provides rich visual and contextual information to reduce ambiguity, as shown in Figure 3a. To learn the relationship between the body and joints, this study considers the 3D positions of the joint nodes as the second information source ${\left(P_{n}^{3D}\right)}_{n=1}^{N}$ (Figure 3b), which can be regarded to be representative of the original joint position, from which the network can extract rich and complex geometric relationships within the human body structure. For learning the relationship between the body and shape, this study considers the shape information to be the third information source ${\left(S_{n}\right)}_{n=1}^{N}$ (Figure 3c), which includes ten parameters such as the height, weight, thinness, and head and body ratio of the human body. The network can then extract rich and complex relationships of the human body and shape from this information. For learning the constraints between the joints of the body, this study considers the geometric descriptor as the fourth source of information G(Δx,Δy,Δz) (Figure 3d), which is motivated by traditional methods based on image structure. It explicitly encodes pairs of relative positions and distances between body parts and reduces the complexity of learning domain prior knowledge, such as relative limb length, joint angle limitations, and symmetry of body parts.

### 3.4. Loss Functions

Let $I={\left(I_{n},P_{n},S_{n}\right)}_{n=1}^{N}$ denote the MPI-INF-3DHP and SURREAL dataset, wherein N denotes the sample indexes. Specifically, $N=\left\{N_{2D},N_{3D}\right\}$, where $N_{2D}$ and $N_{3D}$ are the sample indexes for the 2D and 3D datasets. Each sample (I,P,S) consists of the image I, GT body pose locations P, and GT shape S, where $P={\left\{\left(x^{i},y^{i}\right)\right\}}_{j=1}^{K}$ for the 2D dataset and $P={\left\{\left(x^{i},y^{i},z^{i}\right)\right\}}_{j=1}^{K}$ for the 3D pose dataset. Here, K denotes the total number of body joints, and  j denotes the index of body joints.

#### 3.4.1. Generator Loss

The loss in 3D pose [7] is given by:

(1) $$L_{3D}=\overset{K}{\underset{j=1}{\sum }}\left(\underset{n\epsilon N}{\sum }\|x_{n}^{j}-{\hat{x}}_{n}^{j}\|{}_{2}^{2}+\underset{n\epsilon N}{\sum }\|y_{n}^{j}-{\hat{y}}_{n}^{j}\|{}_{2}^{2}+\underset{n\epsilon N}{\sum }\|z_{n}^{j}-{\hat{z}}_{n}^{j}\|{}_{2}^{2}\right)$$

where (x,y,z) represents the position of the predicted keypoints, and $\left(\hat{x},\hat{y},\hat{z}\right)$ represents the position of the GT keypoints. Here, j denotes the index of body joints.

The loss in 2D pose [7] is given by:

(2) $$L_{2D}=\overset{K}{\underset{j=1}{\sum }}\left(\underset{n\epsilon N}{\sum }\|x_{n}^{j}-{\hat{x}}_{n}^{j}\|{}_{2}^{2}+\underset{n\epsilon N}{\sum }\|y_{n}^{j}-{\hat{x}}_{n}^{j}\|{}_{2}^{2}\right)$$

where (x,y) represents the position of the predicted keypoints, and $\left(\hat{x},\hat{y}\right)$ represents the position of the GT keypoints. Here, j denotes the index of body joints.

The loss in shape [9] is given by:

(3) $$L_{shape}=\overset{K}{\underset{j=1}{\sum }}\left(\underset{n\in N}{\sum }\|s_{n}^{j}-{\hat{s}}_{n}^{j}\|{}_{2}^{2}\right)$$

where (s) represents the predicted shape, and $\left(\hat{s}\right)$ represents the shape of the GT. Here, j denotes the number of body joints. *n* denotes the sample indexes for the datasets, and *N* denotes the total sample number for the datasets.

#### 3.4.2. Adversarial Learning

After pretraining the shape–pose-based generator, the generator and discriminator are optimized. The loss of the discriminator [9] is given by:

(4) $$F_{Dloss}=\underset{n\in N_{3D}}{\sum }\zeta_{cls}\left(D\left(I_{n},E\left(\hat{x_{n}},\hat{y_{n}},\hat{z_{n}},\hat{s_{n}}\right)\right),1\right)+\underset{n\in N_{}}{\sum }\zeta_{cls}\left(D\left(I_{n},E\left(G\left(I_{n}\right)\right)\right),0\right)$$

where $E\left(\hat{x_{n}},\hat{y_{n}},\hat{z_{n}},\hat{s_{n}}\right)$ encodes the pose and shape, $\left(\hat{x},\hat{y},\hat{z}\right)$ represents the position of the GT keypoints, and $\left(\hat{s}\right)$ represents the shape of the GT. $D\left(I_{n},E\left(\hat{x_{n}},\hat{y_{n}},\hat{z_{n}},\hat{s_{n}}\right)\right)\in \left[0,1\right]$ is the classification score of the discriminator for the input image $I_{n}$ and encoding information $E\left(\hat{x_{n}},\hat{y_{n}},\hat{z_{n}},\hat{s_{n}}\right)$. $G\left(I_{n}\right)$ is the 3D information predictor, and the corresponding 3D information can be predicted according to the input image. $\zeta_{cls}$ is the cross-entropy loss, which is defined as:

(5) $$\zeta_{cls}\left(\hat{y},y\right)=-\left(y\log\left(\hat{y}\right)+\left(1-y\right)\log\left(1-\hat{y}\right)\right)$$

## 4. Experimental Analysis

This study conducted experiments to demonstrate 3D human mesh reconstruction learning from multiple annotated databases and a good 3D human reconstruction performance from in-the-wild images.

The GAN is usually trained from scratch by alternately optimizing the generator and discriminator [15,21]. However, for this task, the proposed method enables faster training of the network and better performance using the pre-trained generator (i.e., the pose–shape-based generator).

### 4.1. Experimental Environment and Datasets

The experiments were carried out using a desktop computer running the Ubuntu 16.04.5 operating system and using four Titan 1080Ti GPUs. The CUDA toolkit version 9.2 and cuDNN version 7 were employed, and Python 2.7 and TensorFlow were configured on the system. Training was conducted using six datasets, as described in Table 2.

The experiments were conducted using 2D annotated datasets and 3D datasets. For the 2D annotated datasets, LSP and LSPE provided a total of 11,000 images, among which 10,000 images were used for training and the rest were used for validation. MS COCO provided 124,000 images, from which 83,000 were used for training and 41,000 for testing.

MPI-INF-3DHP was used as the 3D dataset. This dataset was generated in a controlled environment and provided with 3D annotations. It contained 150,000 training images. MoSh data was also used to train the SMPL. Human3.6M has a total of seven subjects, this paper is trained on five subjects (S1, S5, S6, S7, S8) and tested on two subjects (S9, S11).

All images were scaled, and the aspect ratio was preserved such that the diagonal of the tight bounding box was approximately 150 px. The images were randomly translated, scaled, and flipped.

### 4.2. Experimental Setting

**Figure 4:**
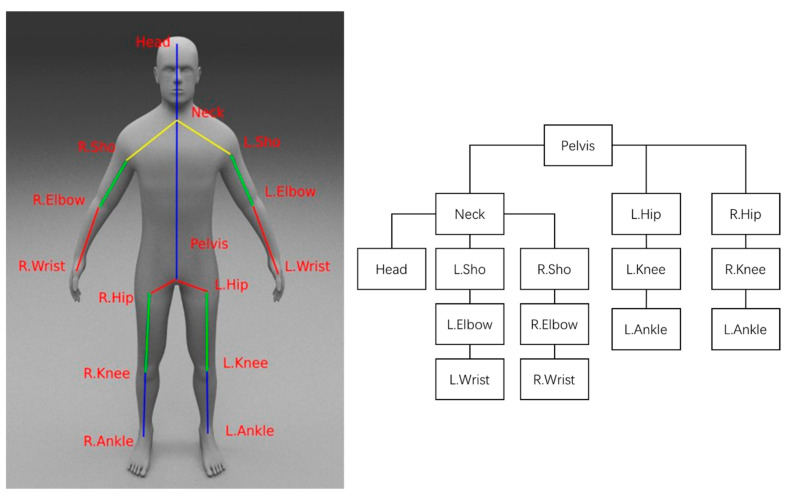
Human Skeleton Model with 15 Joints.

The human body is a highly complex system comprising several limbs and joints. Estimating the 3D joint positions realistically is a daunting task even for humans. In this study, a model-based approach was adopted to construct a mannequin and introduce prior information to enforce constraints. Figure 4 shows a human skeleton model with 15 joints, which was used to conduct the experiments. The 15 joints can be represented by a tree-structured representation with the pelvis as the root node. Sho refers to Shoulder, Elb refers to Elbow, Ank refers to Ankle, R signifies Right, and L signifies Left.

The experimental parameters are shown in Table 3. The input image size was 256 × 256 pixels, and the output was a 3D mesh model. For the encoding 2D pose module, the heatmap representing the 2D pose ${\left(P_{n}^{2D}\right)}_{n=1}^{N}$ was used. The resolution of all input images was adjusted to 256×256 pixels. The network predicted one channel for each body joint (the total number of joints in the human body was k=15).

For the encoding shape module, the resolution of all input images was also adjusted to 256×256 pixels. The network output was 128×4×4. This study vectorizes the output of this network and adds three fully connected layers (fc1(2048,1024), fc2(1024,512), and fc1(521,10)) to produce the parameters ${\left(S_{n}\right)}_{n=1}^{N}$.

### 4.3. Experimental Results

The various challenges presented by the human posture and shape estimation tasks required several assessment indicators. Consequently, even for methods that used the same dataset, a fair comparison between the methods in question was not possible because the processes of training and evaluation were different.

#### 4.3.1. Feature Extraction in Generator

**Figure 5:**
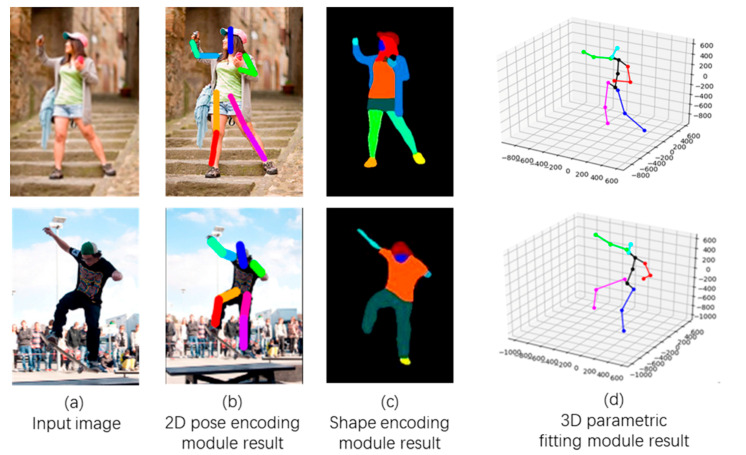
Results of Feature Extraction in Generator.

Figure 5 shows the results of the feature extraction in the generator, this part being the intermediate result of the proposed method, where Figure 5a is the input image, Figure 5b is the output of the 2D pose encoding module (which is the 2D pose data of the figure in the input image), Figure 5c is the output of the shape encoding module (which is the shape data of the figure in the input image), and Figure 5d is the 2D pose data of the figure (based on the 3D pose data obtained from the 2D image). The image in the first row of Figure 5 was sourced from the Internet, and the data in the second row were from the MS COCO [24] dataset. It can be seen here that the proposed method extracts the 2D and 3D poses and shapes of the features in the input images significantly well, providing good data for the next processing step.

#### 4.3.2. Human Mesh Generation 

**Figure 6:**
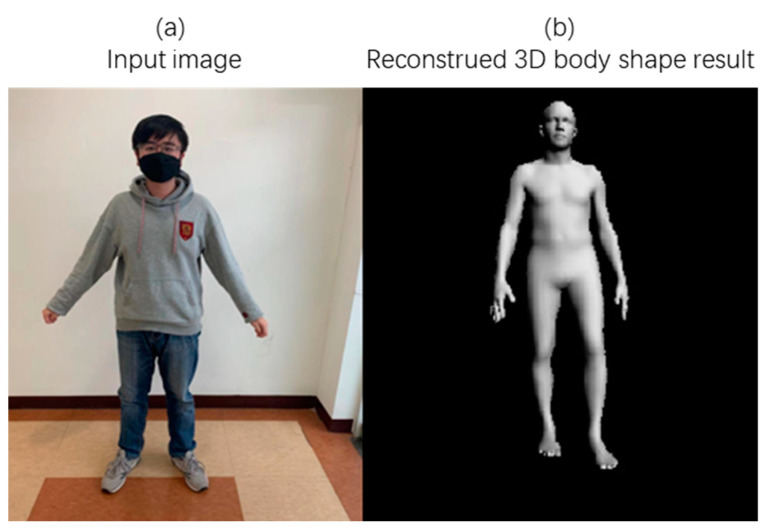
Result of Human Mesh Generation with the Proposed Method using a Single Image.

Figure 6a shows the input image (from the field image, not included in the database), and Figure 6b is the result of the 3D body model generated by the proposed method using the input image. It can be seen here that the proposed method extracts the 3D pose and shape of the figure in the input image, and the generated model accurately reproduces the body pose and shape of the figure.

**Figure 7:**
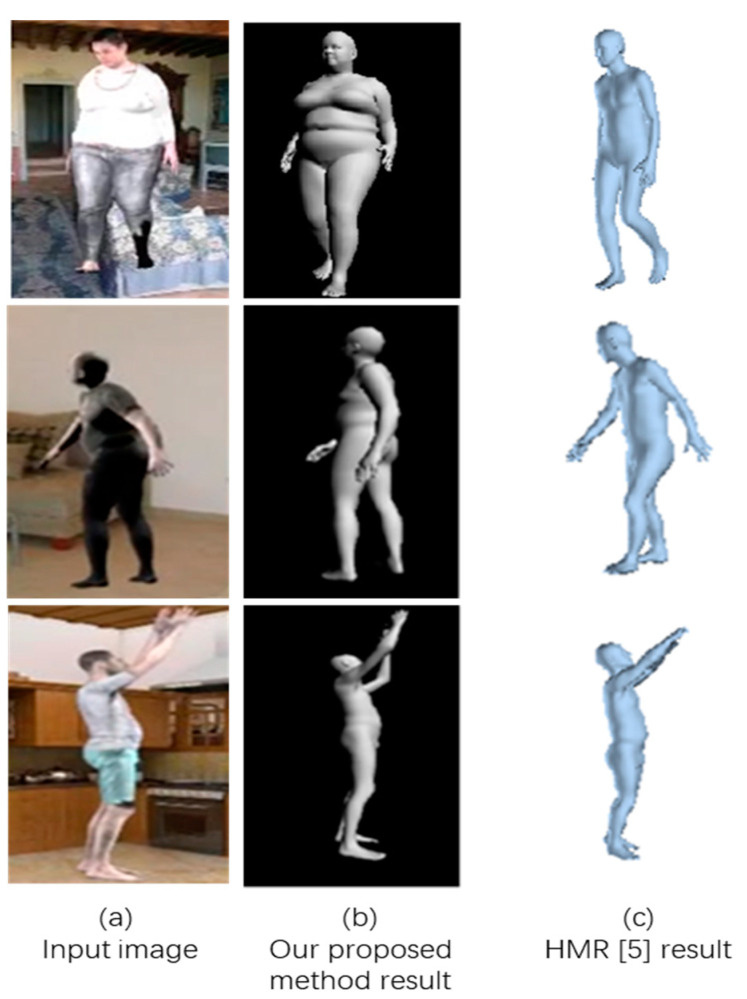
Comparison Result using SURREAL Dataset.

Figure 7a is the input image (from the SURREAL database), Figure 7b is the result obtained using the proposed method, and Figure 7c is the result obtained using the HMR method [7]. From Figure 7, we can clearly see that the proposed method more accurately reproduced the shape of the figure.

**Figure 8:**
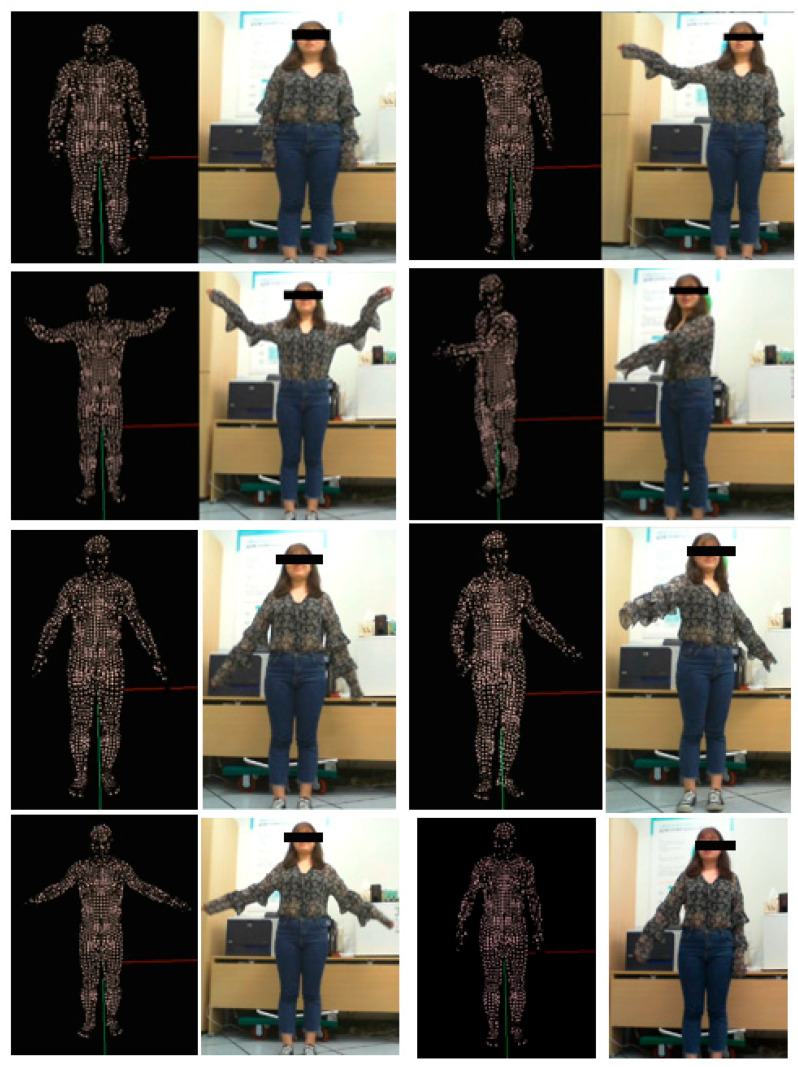
Real-time Human Model Generation from a Video using the Proposed Method.

Figure 8 shows images taken from a video (the participant stood in front of the camera and performed random actions to test the real-time performance of the proposed method). The left section of the figure shows the 3D human body model generated by the proposed method and the right section shows the input image. In order to render quickly and produce results in real time, we used points instead of a mesh. Here, it can be seen that the proposed method was able to accurately and quickly extract the 3D poses and shapes of the feature and generate models and results in real time. Because there are no ground truth meshes for this practical test, we visualize the results in different frames to show that method proposed in this paper can restore the meshes accurately in real time, even when the participant performs various actions.

### 4.4. Component Evaluation 

We evaluated the proposed method using pose and segmentation evaluation methods. The various challenges in the human posture and shape estimation tasks required several assessment indicators. As the database could be divided into 2D and 3D databases, the evaluation criteria should also be divided into 2D and 3D categories. This study chose to employ current, mainstream methods to evaluate the 3D joint errors. Most common evaluations report the mean per joint position error (MPJPE). The per joint position error is the Euclidean distance between the GT and the prediction for a joint; the MPJPE is the mean of the per joint position error for all k joints (in this study, k = 15). Calculations were performed after aligning the root joints (typically the pelvis) of the estimated and the GT 3D pose.

#### 4.4.1. Pose Evaluation 

This study used currently popular evaluation criteria for the posture evaluation, considering the dataset, namely the 3D error [29]. The 3D error is the mean squared distance in 3D (measured in millimeters) between the set of virtual markers corresponding to the joint centers and the limb ends, as described in Equation (6):

(6) $$F_{3Derror}\left(x,\hat{x}\right)=\frac{1}{M}\overset{M}{\underset{i=1}{\sum }}\|m_{i}\left(x\right)-m_{i}\left(\hat{x}\right)\|$$

where x represents the ground truth pose, $\hat{x}$ refers to the estimated pose, M is the number of virtual markers, and $m_{i}\left(x\right)$ represents the 3D position of the $i^{\mathrm{th}}$ marker. This evaluation method is also called the MPJPE [28].

The results of pose estimations are shown in Table 4 (MPJPE loss is shown in millimeters). This study evaluated the 3D joint errors on Human3.6M, which was captured in a laboratory environment using the standard 3D pose benchmark. To compare the results fairly, we trained our model on the same dataset [28] utilized by other methods. The results obtained using the proposed method were comparable to those of state-of-the-art methods.

#### 4.4.2. Segmentation Evaluation

For shape evaluation, we evaluated the acquired six body part segmentation results to obtain meaningful performance scores. We evaluated our method using the F1-Score [30], which is the harmonic mean of precision and recall. The advantage of the F1-Score is that it takes both false positives (due to precision) and false negatives (due to recall) into account, as shown in Equation (7) [30]:

(7) $$F1=\frac{2}{\frac{1}{precision}+\frac{1}{recall}}=\frac{2\times \left(precision\times recall\right)}{precision+recall}$$

Table 5 depicts the foreground and part segmentation (6 parts + bg) on the LSP. To compare the results fairly, we trained our model on the same dataset [22,23] utilized by other methods. In the table, FB seg denotes foreground segmentation, which refers to the overall segmentation accuracy for a human. Part Seg refers to partial segmentation, which consists of six body parts: front, torso, left and right knees, and left and right arms. It provides a reasonable approximation of the overall consistency of a fit, although this representation is coarse. It takes into account the shape of the body and not just the keypoints. The segmentation accuracies of different studies were shown using the projection of the 3D shape estimate on the image. Higher average accuracies and F1-scores signify better results. It can be seen that the results obtained via the proposed method were comparable to those of state-of-the-art methods. Previous research [7,30] has shown that the prediction of human posture through deep learning is valid and credible. Although previous studies have achieved more accurate posture estimations, this study improves both the accuracy of posture estimation and the accuracy of the body shape. In order to facilitate the comparison of experimental data, the evaluation method is kept consistent with other methods. The obtained results are shown in Table 5. Notably, the segmentation of the SMPL mesh part definition is not quite the same as that of the annotation that restricts the highest possible precision to less than 100%.

## 5. Conclusions and Future Work

This paper proposes a human body mesh reconstruction method that can generate a 3D human body mesh from a single image. Compared to other methods, this method uses an in-the-wild image dataset annotated with 2D keypoints and semantic segmentation to reduce the size of 3D annotated datasets. The pose and shape in the RGB image data are extracted via two network meshes. The pose and shape parameters are then fed to the fitting 3D parametric mesh to obtain the 3D parameters. Further, a discriminator is used to identify whether the mesh conforms to reality. Unlike other methods that focus only on predicting 3D human pose [7,15,21], the method proposed herein more accurately recovers the 3D pose and shape of the human body from a single image, while requiring a shorter prediction time. The experimental results prove that the restored 3D pose in this study achieves an error of just 79.37 mm, which is comparable to those of current state-of-the-art methods. The proposed method also achieves a higher accuracy of 92.1% and a shorter predicting time, with 34 frames being processed per second. Moreover, through a reduction in the use of 3D databases, the proposed method significantly increases the amount of data that can be used for training, facilitating easier training of the network and eliminating the problem of insufficient 3D training datasets. Moreover, several 3D databases were used, enabling the data to be more realistic and reliable. With the ability to recover the pose and shape of a human body accurately, the method can be utilized to generate various 3D human meshes with only single view images, which facilitates 3D modeling applications. The 3D meshes can be utilized in smart cities to simulate pedestrians and customers to reduce the modeling cost. However, the method proposed herein cannot recover the clothes of the figure in real time and can only add texture to the 3D model through other tools. In the future, the quantity of training data will be expanded, the experiment will be optimized, and the performances of the generator and discriminator will be enhanced. In addition, a method to automatically add clothes to the restored meshes will also be proposed.

## Figures and Tables

**Table 1:** Features of Different Methods.

	Features	Mesh Generation	Pose Estimation	Multi-Person Pose Estimation	Real-Time Estimation	In-the-Wild Image Support
Approach						
3d Human Pose Estimation by GAN [9]		No	Yes	No	Not Mentioned	Yes
HMR [7]		Yes	Yes	No	Yes	Yes
Bio-LSTM [6]		Not Mentioned	Yes	Yes	Not Mentioned	Not Mentioned
Proposed Method		Yes	Yes	No	Yes	Yes

**Table 2:** Introduction to Dataset.

Dataset Name	Contents	Skeletal Annotation Dimension
Leeds Sports Pose (LSP) [22] & Leeds Sports Pose Extended (LSPE) [23]	10,000 images are included in these two datasets, collected from Flickr using multiple tags such as “parkour”, “gymnastics”, and “athletics”. The poses included are challenging to estimate.	2D
MS COCO [24]	COCO is a large image dataset designed for object detection, segmentation, person keypoint detection, stuff segmentation, and caption generation. This dataset contains photos of 91 object types that can be easily recognized.	3D
MPI-INF-3DHP [25]	This dataset was generated in a green-screen studio with 14 cameras used for recording. It has segmentation masks available for background, chair, and upper and lower body clothing.	3D
MoSh [26]	The mocap lab in the basement of Wean has 12 Vicon infrared MX-40 cameras, which can record 4-MP resolution images at 120 Hz. For generating this dataset, the cameras were placed in the center of the room and covered an approximate rectangular area of 3 m × 8 m. Only motions conducted within this rectangle can be captured.	3D
SURREAL [27]	This is a new large-scale dataset containing synthetically generated but realistic images of people rendered from 3D sequences of human motion capture data.	3D
Human3.6M [28]	a standard 3D pose benchmark captured in a lab environment	3D

**Table 3:** Experimental Parameters.

Parameter Name	Parameters and Dimensions/Size
Input image size	256 × 256
Output	3D mesh
Joints Number	15
Mini-batch Size	64
Learning Rates (Generator)	1 × 10^−5^
Learning Rates (Discriminator)	1 × 10^−4^
Epochs	55

**Table 4:** Comparison of Pose Estimation Results.

Method	MPJPE
Proposed Method	**79.37**
VNect [19]	80.5
HMR [7]	87.97
Tome et al. [15]	88.39
HMR Unpaired [7]	106.84
Deep Kinematic Pose [21]	107.26

**Table 5:** Comparison of Shape Estimation Results.

Method	FB Seg		Part Seg		Run Time
	Acc	F1	Acc	F1	
Decision Forests [30]	86.60	0.80	82.32	0.51	0.15 s
HMR [7]	91.67	0.87	87.12	0.60	0.05 s
HMR Unpaired [7]	91.30	0.86	87.00	0.59	0.04 s
Proposed Method	**92.10**	**0.88**	**88.37**	**0.67**	**0.03 s**

## Data Availability

Data available in a publicly accessible repository. The data presented in this study are openly available in [Leeds Sports Pose (LSP); Leeds Sports Pose Extended (LSPE), MS COCO, MPI-INF-3DHP, MoSh, SURREAL, Human3.6M] at [10.5244/C.24.12, 10.1109/CVPR.2011.5995318, 10.1007/978-3-319-10602-1_48, 10.1109/3DV.2017.00064, 10.1145/2661229.2661273, 10.1109/CVPR.2017.492, 10.1109/TPAMI.2013.248], reference number [22,23,24,25,26,27,28].
